# A simple and efficient method for poly-3-hydroxybutyrate quantification in diazotrophic bacteria within 5 minutes using flow cytometry

**DOI:** 10.1590/1414-431X20165492

**Published:** 2017-01-16

**Authors:** L.P.S. Alves, A.T. Almeida, L.M. Cruz, F.O. Pedrosa, E.M. de Souza, L.S. Chubatsu, M. Müller-Santos, G. Valdameri

**Affiliations:** 1 Departamento de Bioquímica e Biologia Molecular, Universidade Federal do Paraná, Curitiba, PR, Brasil; 2 Departamento de Análises Clínicas, Universidade Federal do Paraná, Curitiba, PR, Brasil

**Keywords:** Flow cytometry, Nile red, Poly-3-hydroxybutyrate, Herbaspirillum seropedicae, Azospirillum brasilense

## Abstract

The conventional method for quantification of polyhydroxyalkanoates based on whole-cell methanolysis and gas chromatography (GC) is laborious and time-consuming. In this work, a method based on flow cytometry of Nile red stained bacterial cells was established to quantify poly-3-hydroxybutyrate (PHB) production by the diazotrophic and plant-associated bacteria, *Herbaspirillum seropedicae* and *Azospirillum brasilense*. The method consists of three steps: i) cell permeabilization, ii) Nile red staining, and iii) analysis by flow cytometry. The method was optimized step-by-step and can be carried out in less than 5 min. The final results indicated a high correlation coefficient (R^2^=0.99) compared to a standard method based on methanolysis and GC. This method was successfully applied to the quantification of PHB in epiphytic bacteria isolated from rice roots.

## Introduction

Polyhydroxyalkanoates (PHAs) are biodegradable polymers that can provide an environmentally friendly alternative to replace petroleum-based plastics (1). Poly-3-hydroxybutyrate (PHB) is the most abundant naturally-occurring polyester produced by bacteria in response to carbon oversupply and other nutrient limitations, such as low nitrogen levels (2). PHB is stored as cytoplasmic granules with a diameter ranging from 0.2 to 0.5 µm, and can provide carbon and energy for the bacteria under certain conditions (3).

The initial studies concerning PHAs detection were based on gravimetric and infrared spectroscopy methods, and the earliest research in PHB quantification was performed using gas chromatography (GC) (3). Although several methods have been described for this purpose, such as HPLC (4), fluorescent dyes (5), ionic chromatography, and enzymatic methods (6), GC is still the preferred standard method for PHB quantification, showing high sensitivity, accuracy, and reproducibility. However, there are three major drawbacks associated with GC-based methods: i) the use of hazardous solvents at high temperature, ii) a long time requirement for sample processing, and iii) the need for large amounts of bacterial cells. Thus, there is a need for alternatives to GC for PHB quantification.

In the early 1980's, Nile red [9-diethylamino-5H-benzo(α)phenoxazine-5-one] was described as a promising fluorescent dye for detection of intracellular lipid droplets by flow cytometry in aortic smooth muscle cells and on cultured peritoneal macrophages (7). In the 1990's, a method based on Nile red (NR) staining was applied for the detection of PHB production in *Alcaligenes eutrophus* (8) and *Ralstonia eutropha* H16 (5). To date, several protocols established for NR staining and PHB quantification by flow cytometry in *Saccharomyces cerevisae*, *Cupriavidus necator* (9), *Synechocystis* sp. strain PCC6803, *Escherichia coli* (10), and three *Pseudomonas sp.* (11) have been reported. The optimization of a flow cytometry protocol for the quantification of PHB requires the determination of conditions for efficient cell permeabilization and optimal NR concentration, parameters that are dependent on cell type, membrane properties and bacterium size.

*Herbaspirillum seropedicae* and *Azospirillum brasilense* are plant growthpromoting diazotrophic bacteria (12,13). While there are several indications that PHB plays important roles in nitrogen fixation and plant-bacteria interactions (14–17), the real significance of PHB for bacteria during plant-colonization remains unknown. The development of techniques allowing PHB quantification in small volumes and low cell numbers will allow data collection in restrictive conditions – such as in bacteria colonizing roots and other plant tissues – and will contribute to the determination of the true role of PHB in plant-bacteria association. In this study, we describe the optimization and validation of a simple, fast and accurate method for the quantification of PHB production in *H. seropedicae* and *A. brasilense*, based on NR staining and flow cytometry.

## Material and Methods

### Reagents and buffers

Phosphate-buffered saline (PBS) containing 137 mM NaCl, 2.7 mM KCl, 8 mM Na_2_HPO_4_ and 1.4 mM KH_2_PO_4_ at pH 7.2 NR (Sigma Aldrich, USA) was dissolved in DMSO to a final concentration of 3.14 mM (1 mg/mL) and kept in the dark. NR was further diluted in different buffers as indicated in the Figure legends. TBAC buffer [PBS containing 1 mM EDTA and 0.01% (v/v) Tween 20] was used to avoid the formation of bacterial aggregates that could potentially perturb light-scattering and fluorescence signals in flow cytometric analysis. TSE buffer contains 10 mM Tris-HCl pH 7.5, 20% (wt/vol) sucrose and 2.5 mM EDTA. All other reagents were commercial products of the highest purity grade available.

### Bacterial strains and growth conditions

*H. seropedicae* strain SmR1 (wild type) (18) and strain Δ*phaC1*, an SmR1 mutant deficient in PHB synthesis, previously described as Δ*phbC1* (19), were cultivated in NFbHP-malate medium containing 0.5% of DL-malic acid and 20 or 5 mM of NH_4_Cl. *A. brasilense* strain FP2 (20) and *A. brasilense* Sp7 mutant strain *phbC* (21) were cultivated in NFbHP-lactate medium containing 0.5% of DL-lactic acid and 20 or 5 mM of NH_4_Cl. *A. brasilense* FP2 is a spontaneous mutant strain from *A. brasilense* Sp7 resistant to nalidixic acid and streptomycin (20). Antibiotics were added to the growth media in the following concentrations: streptomycin (80 µg/mL) for *H. seropedicae*, streptomycin (80 µg/mL) and nalidixic acid (10 µg/mL) for *A. brasilense* strain FP2, and kanamycin (100 µg/mL) for *A. brasilense* Sp7 mutant strain *phbC*.

### PHB quantification by gas chromatography

The bacterial PHB amount was determined by acid methanolysis followed by GC coupled to a flame-ionization detector as previously described (22). Methanolysis was performed with 5–10 mg of lyophilized bacteria in 2 mL of chloroform and 2 mL of methanol containing 15% sulphuric acid in borosilicate glass tubes with screw caps. For each reaction, 0.5 mg of benzoic acid was added as internal standard. Reaction mixtures were incubated at 100°C for 3.5 h in a dry-heating block. After cooling, 1 mL of distilled water was added and the tubes were vortexed for 60 s. The upper aqueous phase was removed and the lower organic phase containing the resulting 3-hydroxybutyric methyl ester (Me-3-HB) was dried with Na_2_SO_4_ and analyzed by GC in a 450 GC chromatograph (Varian, Netherlands) equipped with a CP-Sil-5 CB column (10 m×0.53 mm ID). Argon was used as carrier gas at 0.9 mL/min. The injector was set at 250°C and the detector at 275°C. The oven temperature program was: initial temperature 50°C for 2 min, then from 50°C up to 110°C at a rate of 20°C/min and finally up to 250°C at a rate of 20°C/min. The PHB amount in each sample was normalized by the weight of the lyophilized bacteria and expressed as a percentage of PHB/cell dry weight.

### PHB quantification by flow cytometry

Flow cytometry experiments were performed in a BD Accuri C5^®^ Flow Cytometer (USA) equipped with a 488-nm laser for fluorescence excitation. For each sample, 100,000 events were acquired, and the median fluorescence intensities were obtained from histograms of FL2-H 585/40 nm channel. Flow cytometry calibration was performed using spherothech 8-peak beads (BD Accuri^™^, USA) according to the manufacturer's recommendations and instructions.

### Optimization of the flow cytometric protocol for PHB quantification

For all steps of the optimization process, an aliquot of 100 µL (∼10^6^–10^7^ cells/mL) of the cell culture was centrifuged for 1 min at 13,400 *g* at room temperature, the supernatant solution was discarded and the cell pellet was treated according to each specific condition. For all conditions, after staining with NR, the cells were collected by centrifugation for 1 min at 13,400 *g* and resuspended in TBAC buffer for analysis by flow cytometry. During the optimization process the following steps were carried out in order of description:

1. Cell permeabilization conditions: the cell pellet was resuspended in 1 mL of each of the evaluated membrane permeabilization solutions (TBAC containing 30% of ethanol, TBAC containing 0.1% of Triton X-100 and TSE buffer). The bacterial suspensions were stained with NR (9.42 µM) for 5 min and analyzed by flow cytometry.
2. Optimization of the ethanol concentration for cell permeabilization: the cell pellet was resuspended in 1 mL of TBAC buffer containing increasing concentrations of ethanol (up to 70%). After 5 min of ethanol exposure, cells were stained with NR (9.42 µM) for 5 min.
3. Bacterial cell permeabilization time: the cell pellet was resuspended in 1 mL of TBAC buffer containing 50% of ethanol and incubated up to 30 min. After ethanol exposure, cells were stained with NR (9.42 µM) for 5 min.
4. Optimization of NR staining: the cell pellet was resuspended in 1 mL of TBAC buffer containing 50% of ethanol for 1 min. After ethanol exposure, cells were stained with NR (9.42 µM) for up to 30 min.
5. Determination of the optimal NR concentration: the cell pellet was resuspended in 1 mL of TBAC buffer containing 50% of ethanol for 1 min. After ethanol exposure, cells were stained with NR (0 to 500 µM) for 1 min.
6. Fluorescence stability: the cell pellet was resuspended in 1 mL of TBAC buffer containing 50% of ethanol for 1 min. Cells were subsequently stained with NR (31.25 µM) for 1 min, centrifuged (1 min at 13,400 *g*), and resuspended in TBAC buffer for analysis by flow cytometry. The NR fluorescence was monitored during 90 min by flow cytometry. In addition, samples were stored at 4°C in permeabilization solution (TBAC buffer containing 50% of EtOH), until analysis.

### PHB quantification by flow cytometry using NR fluorescence

The optimized protocol for the quantification of PHB by flow cytometry is described as follows: an aliquot of 100 µL (∼10^6^–10^7^ cells/mL) of a bacterial culture is centrifuged for 1 min at 13,400 *g*. The supernatant solution is discarded and the cell pellet is resuspended in 1 mL of TBAC containing 50% of ethanol. After 1 min of incubation, samples are stained with 31.25 µM of NR for 1 min in the dark, centrifuged 1 min at 13,400 *g*, and the supernatant solution discarded. The pellet is then resuspended in 1 mL of TBAC solution and immediately analyzed by flow cytometry.

### PHB staining for fluorescence microscopy

The same optimized protocol to prepare bacterial cells stained with NR for flow cytometry was applied to prepare cells for fluorescence microscopy. The non-optimized protocol (9) differed from the optimized one mainly in the ethanol (30%) and NR concentration (9.42 µM). The fluorescent images were obtained using the Axio Imager Z2 microscope (Carl Zeiss, USA), equipped with the scanning platform Metafer 4 and CoolCube 1 camera (Metasystems, USA) magnifying 100 times.

### PHB production by epiphytic rice bacteria

Rice experiments were performed according to Valdameri et al. (23). PHB measurements in the epiphytic bacterial populations were performed in bacteria detached from plants 7 days after inoculation. Bacteria were removed from roots by vortexing for 1 min in 1 mL of TBAC containing 50% of ethanol. The suspension was stained with NR following the optimized protocol.

## Results

### Screening for permeabilization solutions

To determine the best cell permeabilization solution to stain *H. seropedicae* strain SmR1 and *A. brasilense* strain FP2 with NR, we initially compared three different conditions: i) TBAC containing 30% of ethanol, ii) TBAC containing 0.1% of Triton X-100, and iii) TSE buffer. TBAC buffer was previously applied to determine bacterial cell concentration by flow cytometry (23).

**Figure 1:**
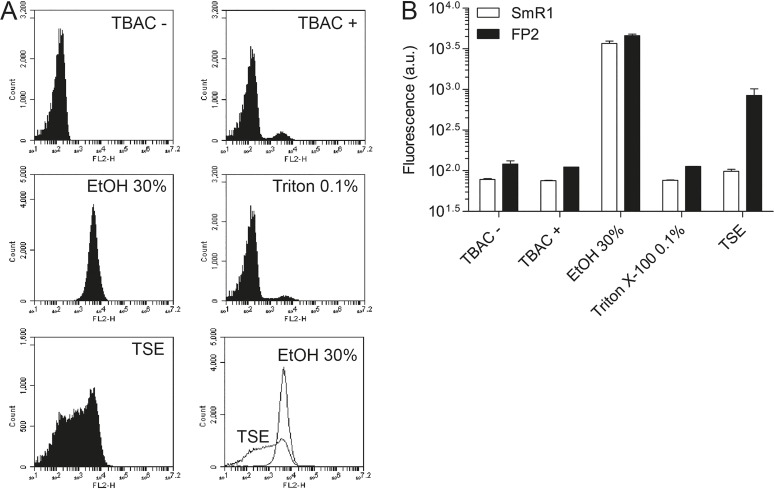
Screening for cell permeabilization solutions. Bacteria were grown at an OD_600_ of 1.2, 0.1 mL was centrifuged (1 min at 13,400 *g*), and resuspended in 1 mL of each solution evaluated. The bacterial suspensions were stained with NR (9.42 µM) during 5 min, and analyzed by flow cytometry. *A*, Histograms are representative of *A. brasilense* strain FP2. Conditions: TBAC- corresponds to Nile red (NR) non-stained samples, used as a blank. All other samples were stained with NR. TBAC+ corresponds to non-permeabilized samples in TBAC. EtOH 30% corresponds to samples in TBAC containing 30% of ethanol. Triton 0.1% corresponds to samples in TBAC containing 0.1% of Triton X-100. TSE corresponds to samples in TSE buffer. TSE/EtOH 30% corresponds to overlay of two histograms, TSE and EtOH 30%, respectively. *B*, Fluorescence data (arbitrary units, a.u.) are reported as means±SD of 3 independent experiments with *H. seropedicae* strain SmR1 and *A. brasilense* strain FP2, by using the median fluorescence intensity values in the FL2-H channel.

As shown in Figure 1A and B, intracellular fluorescence of samples stained with NR in TBAC (TBAC+) or TBAC containing detergent (0.1% triton X-100), did not differ from non-stained (TBAC-) samples. In addition, a sucrose-buffer (TSE) produced only a partial permeabilization effect. The representative histograms of *A. brasilense* strain FP2 permeabilized with TSE buffer clearly showed a heterogeneous cell population (Figure 1A). The same heterogeneous distribution was observed with the *H. seropedicae* strain SmR1 treated with TSE buffer (data not shown). TBAC buffer containing 30% of ethanol (EtOH) permeabilized both *H. seropedicae* strain SmR1 and *A. brasilense* strain FP2, producing a single peak of higher fluorescence, denoting a homogeneous and full membrane permeabilization.

### Optimization of ethanol percentage for cell permeabilization

**Figure 2:**
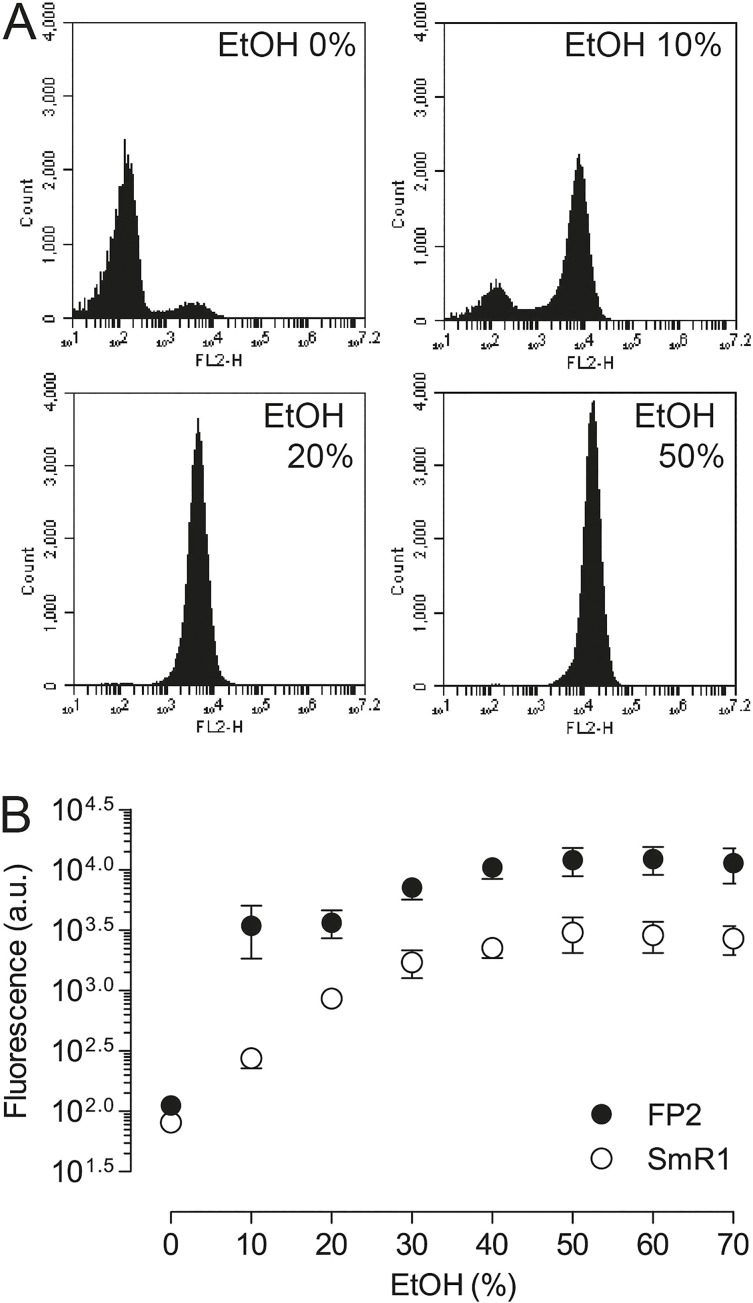
Optimization of ethanol (EtOH) percentage in TBAC buffer for cell permeabilization. Bacteria were grown at an OD_600_ of 1.2, 0.1 mL was centrifuged (1 min at 13,400 *g*), and resuspended in 1 mL of TBAC buffer containing different percentages of EtOH (0-70%). After 5 min of EtOH exposure, cells were stained with Nile red (9.42 µM) during 5 min, centrifuged (1 min at 13,400 *g*), and resuspended in TBAC buffer for analysis by flow cytometry. *A*, Histograms are representative of *A. brasilense* strain FP2 using different percentages of EtOH in TBAC buffer, as indicated. *B*, Fluorescence data (arbitrary units, a.u.) are reported as means±SD of 3 independent experiments with *H. seropedicae* strain SmR1 and *A. brasilense* strain FP2, by using the median fluorescence intensity values in the FL2-H channel.

Since TBAC buffer containing 30% of ethanol permeabilized both bacteria, the effect of ethanol concentration on NR staining was evaluated. Fluorescence histograms of *A. brasilense* strain FP2 reveal that an increase in ethanol concentration increased the amount of permeabilized cells. Indeed, for both bacteria, TBAC buffer containing 50% of ethanol was the best condition for permeabilization considering the increase in NR fluorescence, and a single distinct peak (Figure 2A and B).

### Permeabilization time, Nile red exposure and optimal concentration

**Figure 3:**
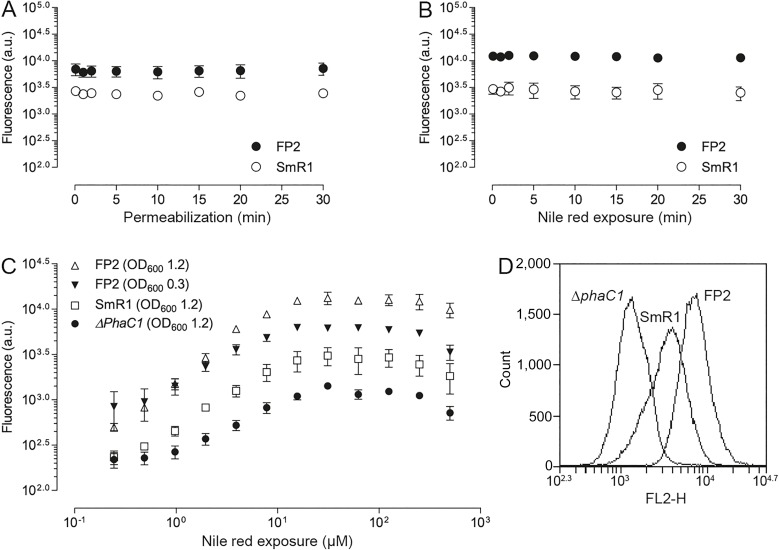
Incubation time required for cell permeabilization and Nile red (NR) staining. Bacteria were grown at an OD_600_ of 1.2, 0.1 mL was centrifuged (1 min at 13,400 *g*), and resuspended in 1 mL of TBAC buffer containing 50% of EtOH. *A*, Exposure with EtOH during 0 to 30 min and cells stained with NR (9.42 µM). *B*, After 1 min of exposure in TBAC buffer containing 50% of EtOH, the bacterial suspensions were incubated with NR (9.42 µM) during 0 to 30 min. *C*, After 1 min of exposure of cells in TBAC buffer containing 50% of EtOH, the bacterial suspensions were stained with NR (0 to 500 µM) during 1 min, centrifuged (1 min at 13,400 *g*), and resuspended in TBAC buffer for analysis by flow cytometry. Fluorescence data (arbitrary units, a.u.) are reported as means±SD of 3 independent experiments with *H. seropedicae* strain SmR1 and *A. brasilense* strain FP2, by using the median fluorescence intensity values in the FL2-H channel. *D*, Histogram overlay of three samples, a mutant strain of *H. seropedicae*, Δ*phaC1*, *H. seropedicae* strain SmR1, and *A. brasilense* strain FP2, using the optimized concentration of NR (31.25 µM).

Cells were incubated in TBAC buffer containing 50% of ethanol up to 30 min before NR staining. As shown in Figure 3A, NR fluorescence levels were similar regardless the incubation time. Therefore, 1 min of permeabilization was used in further experiments, allowing the manipulation of five samples simultaneously. A range of NR incubation periods from 0 to 30 min was also evaluated, and the results showed a similar pattern observed previously for the permeabilization time experiments, with no significant variation in fluorescence values for all sampling times (Figure 3B). Based on these results, the NR time exposure was established as 1 min. It is noteworthy that both *H. seropedicae* strain SmR1 and *A. brasilense* strain FP2 showed the same behavior, allowing the use of the same protocol for both species.

To determine the optimal NR concentration to stain cells with higher fluorescence values, a range from 0 to 500 µM was assayed. Since NR is not a PHB-specific dye, it was necessary to measure the NR fluorescence background in non-PHB producing cells. The *H. seropedicae* SmR1 derived mutant Δ*phaC1* defective in PHB production was tested against the wild type SmR1, both at OD_600_ 1.2. For *A. brasilense* strain FP2, the wild type at low (0.3) and high (1.2) OD_600_ were compared. These conditions were selected based on our GC data (not shown) that have shown a high production of PHB at OD_600_ 1.2 for both wild type strains, whereas both *H. seropedicae* Δ*phaC1* mutant strain and *A. brasilense* strain FP2 at OD_600_ 0.3 did not produce PHB, as detected by the GC method. The optimal NR concentration was 31.25 µM for both bacteria (Figure 3C and D).

### Fluorescence emission stability

**Figure 4:**
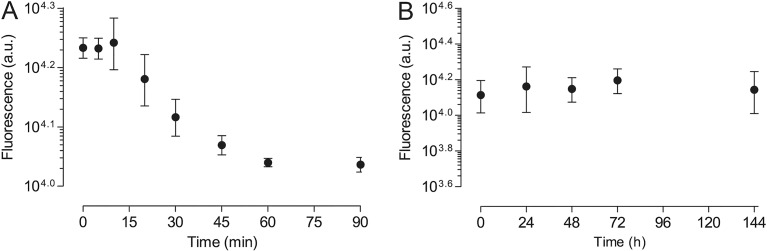
Nile red (NR) fluorescence stability. *A. brasilense* strain FP2 was grown until reaching OD_600_ of 1.4. PHB measurements were performed using the optimized procedure, as described in the Materials and Methods. *A*, After resuspension of cells in TBAC buffer, NR fluorescence was monitored during 90 min by flow cytometry. *B*, Samples were stored at 4°C in permeabilization solution (TBAC buffer containing 50% of EtOH), until analysis, as indicated. For analysis, samples were stained with NR (31.25 µM) during 1 min, centrifuged (1 min at 13,400 *g*), resuspended in TBAC buffer and analyzed immediately by flow cytometry. Fluorescence data (arbitrary units, a.u.) are reported as means±SD of 3 independent experiments with *H. seropedicae* strain SmR1 and *A. brasilense* strain FP2, by using the median fluorescence intensity values in the FL2-H channel.

The last parameter to be optimized was the NR fluorescence stability in TBAC buffer and in non-stained and stored permeabilized samples. The fluorescence emission stability of NR stained samples of *A. brasilense* strain FP2 was monitored for a duration of 90 min (Figure 4A). The NR fluorescence started to decrease 5 min after staining. After 30 min, the fluorescence levels were similar to those of the background level. However, it is worth noting that unstained permeabilized cells can be stored under refrigeration (2 to 8°C) without significant loss of NR staining capacity for up to 5 days (Figure 4B). Therefore, one can store samples prior to NR staining for up to 5 days and then stain cells and capture the emitted fluorescence.

### Flow cytometry *versus* gas chromatography

Several reports have demonstrated a linear correlation between the amount of PHB and the fluorescence emission intensity of NR stained cells. In order to validate the optimized method for PHB quantification by flow cytometry, the standard gas chromatography method was applied to the same bacterial cultures. *H. seropedicae* SmR1 and Δ*phaC1* strains, *A. brasilense* strain FP2, and mutant of *A. brasilense* strain Sp7, identified as *phbC* Sp7, impaired in the production of PHB, were grown in a medium containing 5 and 20 mM NH_4_Cl, since PHB production has been correlated with carbon availability and nitrogen limitation.

**Figure 5:**
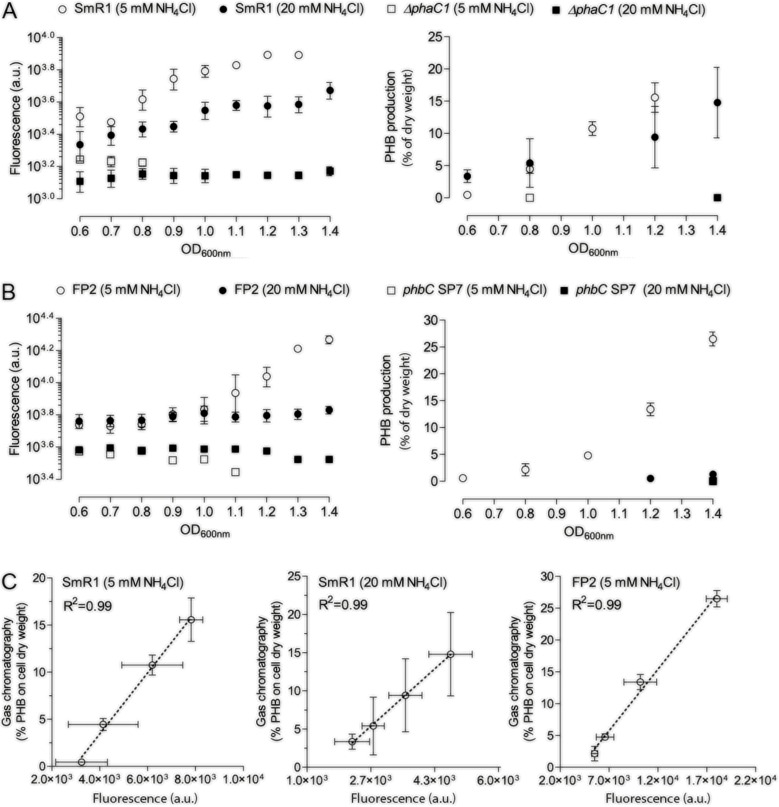
Correlation between flow cytometry and gas chromatography (GC) analysis for PHB quantification. PHB measurements by flow cytometry using the optimized procedure and the standard method based on GC were applied on *H. seropedicae* strain SmR1, a mutant strain of *H. seropedicae*, Δ*phaC1*, *A. brasilense* strain FP2, and a mutant strain of *A. brasilense*, *phbC* SP7, OD_600_ ranging from 0.6 to 1.4 using two NH_4_Cl concentrations in growth medium, as indicated. *A*, *H. seropedicae*. *B*, *A. brasilense*. *C*, Correlation between flow cytometry and GC. Fluorescence data (arbitrary units, a.u.) are reported as means±SD of 3 independent experiments with *H. seropedicae* strain SmR1 and *A. brasilense* strain FP2, by using the median fluorescence intensity values in the FL2-H channel.

The results revealed that the kinetic curves of NR fluorescence as a function of OD_600_ varied among bacteria. As shown in Figure 5A, *H* *. seropedicae* strain SmR1 grown in 5 mM NH_4_Cl produced more PHB as compared to the growth in 20 mM NH_4_Cl, results also confirmed by GC. For *A. brasilense* strain FP2, low NH_4_Cl levels also triggered PHB production; however, *A. brasilense* strain FP2 grown in 20 mM NH_4_Cl did not produce any detectable PHB (results also confirmed by GC; Figure 5B). These data highlighted important differences between PHB accumulation in *H. seropedicae* strain SmR1 and *A. brasilense* strain FP2, since *H. seropedicae* strain SmR1 seems to always produce PHB, even at low OD_600_, whereas *A. brasilense* strain FP2 produces PHB only in OD_600_ 1.0 or higher, with limiting nitrogen concentration in the growth medium. As shown in Figure 5C, flow cytometry and GC present a very high correlation coefficient (R^2^) of 0.99 for both bacteria.

### Fluorescence microscopy analysis

**Figure 6:**
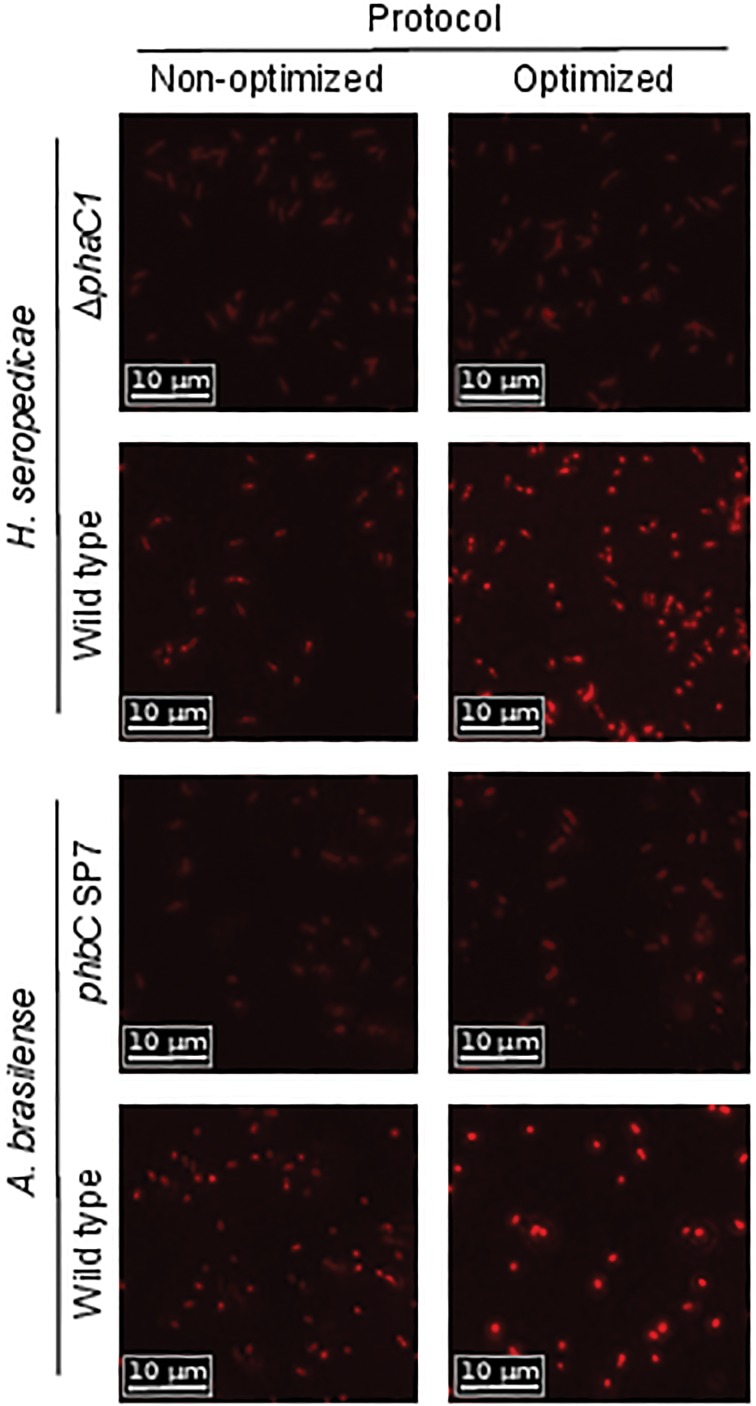
Intracellular PHB detection by fluorescence microscopy using the non-optimized and optimized protocols. *H. seropedicae* strain SmR1, a mutant strain of *H. seropedicae*, Δ*phaC1*, *A. brasilense* strain FP2, and a mutant strain of *A. brasilense*, *phbC* SP7 were grown to OD_600_ of 1.4 using 5 mM NH_4_Cl in growth medium. Fluorescence microscopy analysis was performed using the procedure described in the Material and Methods.

Although this method has been optimized with every precaution possible to ensure high accuracy in flow cytometry determination of PHB, the same protocol can be successfully applied to stain cells for fluorescence microscopy analysis. To confirm this assumption, *H. seropedicae* and *A. brasilense* samples stained with NR using the optimized protocol were analyzed by fluorescence microscopy. In addition, the non-optimized *versus* optimized procedures were compared. Fluorescent micrographs of *H. seropedicae* and *A. brasilense* revealed an increase fluorescence emission intensity in samples stained using the optimized protocol (Figure 6).

### PHB production in epiphytic bacteria analyzed by flow cytometry

**Figure 7:**
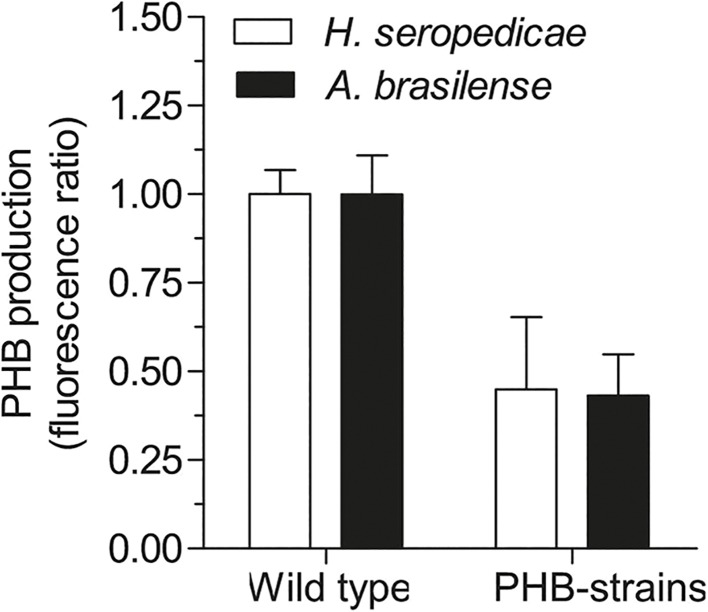
PHB production of epiphytic bacteria analyzed by flow cytometry. PHB measurements were performed using the optimized procedure on rice epiphytic *H. seropedicae* strain SmR1, a mutant strain of *H. seropedicae*, Δ*phaC1*, *A. brasilense* strain FP2, and a mutant strain of *A. brasilense*, *phbC* SP7, 7 days after inoculation, as described in the Material and Methods. Fluorescence ratio data are reported as means±SD of 2 independent experiments performed in triplicate, using the median fluorescence intensity values in the FL2-H channel.

To determine whether this optimized method could be applied to quantify PHB in small-cell-number samples, *H. seropedicae* strain SmR1 and *A. brasilense* strain FP2 cells epiphytically growing on rice roots were detached, stained and PHB was quantified by flow cytometry. After 7 days of rice inoculation, the results showed that both *H. seropedicae* SmR1 and *A. brasilense* FP2 grown epiphytically on rice produced PHB during colonization (Figure 7). This is a first-time demonstration of what can be considered an easy and reliable approach to follow the kinetics of PHB production by epiphytic bacteria. Since the amount of epiphytic cells is usually insufficient to be determined by GC-based methods, the optimized protocol developed in the present work constitutes an important tool to monitor the production of PHB during plant-bacteria interaction, to screen for potential PHB producers among plant-associated bacteria, and in biotechnological studies to evaluate and improve PHB production by bacteria.

## Discussion

For the feasibility of a NR-based method, the dye must cross the bacterial membranes in order to stain the intracellular PHB. Full uptake of fluorescent dyes appears to be critical for a complete intracellular target staining. Besides the hydrophobicity of NR, the bacterial uptake varies widely among different species, essentially due to differences in membrane permeability. Several strategies can be applied to improve the entry of NR, such as those using buffers containing ethanol (9), or sucrose-based buffers, such as the TSE buffer (10).

The sucrose-buffer TSE was successfully applied to permeabilize cells of *Synechocystis* sp. PCC6803 and *Escherichia coli* to NR staining in PHB-producing conditions (10). In the present work the TSE buffer failed to efficiently permeabilize *H. seropedicae* strain SmR1, and produced only a partial effect on *A. brasilense* strain FP2 (Figure 1A and B). The TBAC buffer, on the other hand, containing 50% ethanol was found to be the best permeabilization solution for *H. seropedicae* and *A. brasilense*.

Apparently, the optimal NR concentration can vary among species, and therefore the adjustment of the concentration according to species is a critical step for optimization. For both *H. seropedicae* and *A. brasilense* strains, fluorescence emission increased up to the NR concentration of 31.25 µM, decreasing at higher concentrations of NR (Figure 4A). Despite the very low concentration of NR (0.032 µM) described for both *Saccharomyces cerevisae* and *Cupriavidus necator* (9), most bacteria require higher NR concentrations, as demonstrated for *Synechocystis* sp. strain PCC6803 (3.3 µg/mL – 9.42 µM), *Escherichia coli* (33 µg/mL – 94.2 µM) (10) and *Ralstonia pickettii* AR1 (20 µg/mL – 62.8 µM) (24). Such variations clearly show that protocols must be optimized for each microbe under study before the introduction of NR-fluorescence flow cytometry as a technique to quantify PHB or other kinds of neutral lipids.

Compared to *H. seropedicae* strain SmR1, in all conditions assayed here, *A. brasilense* strain FP2 always produced higher basal fluorescence values (Figures 2, 3, and 5). Two major reasons could explain this observation: i) NR binding to different intracellular lipid droplets, and ii) the difference in size between the *H. seropedicae* strain SmR1 and *A. brasilense* strain FP2, which is almost twice the size. In view of this, we hypothesize that the higher fluorescence emission values observed for *A. brasilense* strain FP2 are probably due to the bacteria size.

Despite the fact that PHA quantification by GC is largely used in microbial PHA research, this methodology is quite laborious and requires hazardous solvent manipulations. The standard method for PHB quantification involving methanolysis followed by GC analysis is a well-established and reproducible technique, however with a main drawback: the long time needed to analyze sample by sample. A typical procedure of methanolysis followed by GC analysis requires 16 h of lyophilization, 5 h of methanolysis and 20 min for GC data acquisition for each sample – at least 250 times longer than our NR optimized protocol using flow cytometry, which was less than 5 min. In addition, our flow cytometry protocol is even faster than other flow cytometry methodologies that require 25 to 50 min to be completed (9,10,24). Another advantage of the NR flow cytometry protocol reported here is the low amount of cells required for analysis, which unlike other methodologies allows one to perform a larger number of experiments in different conditions. While the quantification of other PHAs, such as polyhydroxyhexanoate and polyhydroxyoctanoate, was not tested in this study, we believe our protocol can be successfully adapted to quantify other PHAs.

The methodologies used here, flow cytometry and GC, applied to PHB quantification presented a very high correlation coefficient (R^2^) of 0.99 for both bacteria. This level of correlation is in agreement with methods optimized for other microorganisms, such as *E. coli* (R^2^=0.96) (10), *S. cerevisiae* (R^2^=0.99), and *C. necator* (R^2^=0.99) (9).

In summary, a reliable and relatively fast flow cytometric procedure was developed for PHB quantification in *H. seropedicae* SmR1 and *A. brasilense* FP2 grown in cultures or in cells isolated from grass root surfaces. PHB production can be quantified with accuracy and precision using NR staining, following the six optimized steps detailed in this paper. This protocol has potential to be used in other studies involving PHB metabolism in these and other bacterial species, as well as in quality control of inoculant, since PHB production has been reported as an important feature to maintain the fitness of plant-associated bacteria.
